# Supplementary figures and images for: A single-cell spatiotemporal transcriptomic atlas of mouse prefrontal cortex maps dynamics of intratelencephalic neurons during postnatal development
Source: PLoS Biol. 2026 Jan 12;24(1):e3003594. doi: 10.1371/journal.pbio.3003594 (PMC12818735; doi:10.1371/journal.pbio.3003594)

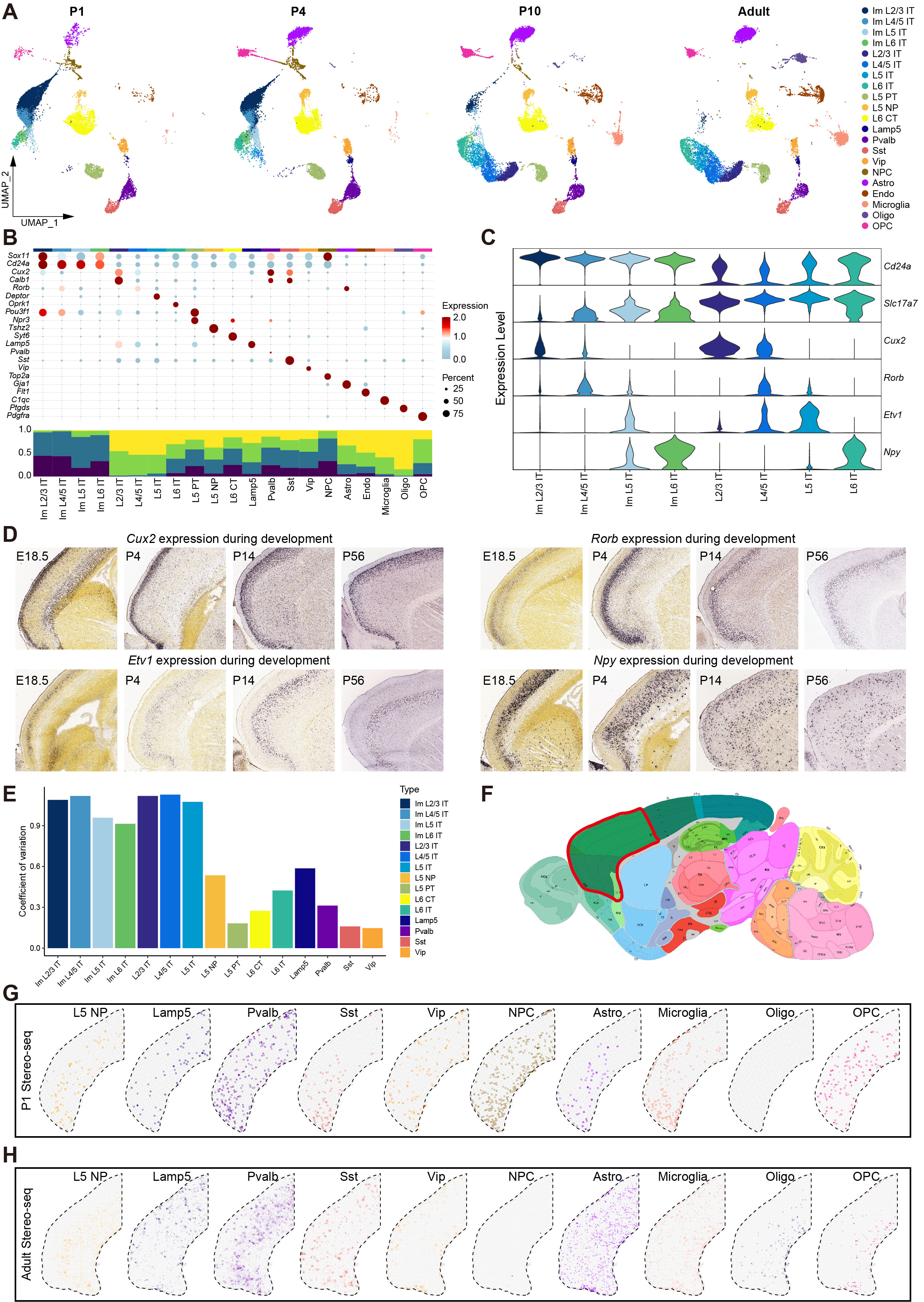

Supplement: S1 Fig — (A) Uniform Manifold Approximation Projection (UMAP) visualization of mouse PFC cell subtypes at different postnatal developmental stages. (B) Dotplot shows the expression patterns of marker genes in cell subtypes (top). Barplot shows the proportion of different time stages in each cell subtype. (C) Violin plot shows the expression patterns of marker genes in IT neuron subtypes. (D) Allen mouse brain ISH images of marker genes at E18.5, P4, P14, and P56. (E) Bar plot shows the coefficient of variation of the changes in the proportion of different neuronal subtypes during development. (F) A full sagittal section of Allen Mouse Brain atlas with the PFC region circled in red color. Allen Mouse Brain Atlas, mouse.brain-map.org and atlas.brain-map.org. (G) Spatial distribution of each cell subtype in mouse PFC from P1 stereo-seq data. (H) Spatial distribution of each cell subtype in mouse PFC from Adult stereo-seq data. (TIF) [file pbio.3003594.s001.tif]

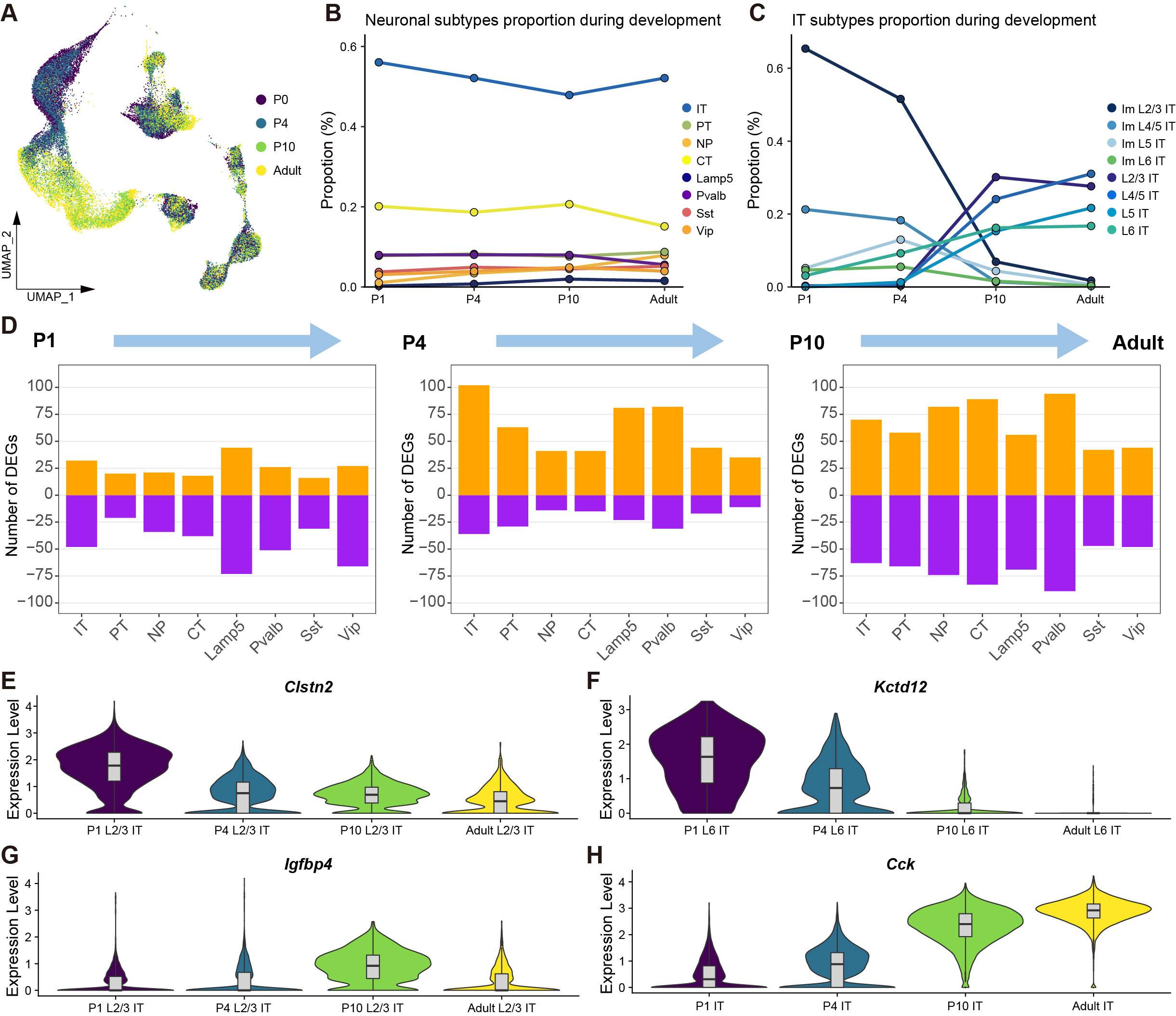

Supplement: S2 Fig — (A) Uniform Manifold Approximation Projection (UMAP) of all neurons in mouse PFC from scRNA-seq, which is colored by time stages. (B) Line plot shows the proportion of each neuronal subtype during postnatal development. IT neurons are merged into one subtype. The data underlying this Figure can be found in S1 Data. (C) Line plot shows the proportion of each IT neuronal subtype during postnatal development. The data underlying this Figure can be found in S1 Data. (D) The number of up-regulated and down-regulated DEGs of each neuronal subtype between different postnatal stages. The neuronal subtypes of each stage are compared with the same neuronal subtypes of the previous stage. Orange, up-regulated genes; purple, down-regulated genes. (E) Violin plot shows the expression level of Clstn2 gene in L2/3 IT neurons at different postnatal stages. (F) Violin plot shows the expression level of Kctd12 gene in L6 IT neurons at different postnatal stages. (G) Violin plot shows the expression level of Igfbp4 gene in L2/3 IT neurons at different postnatal stages. (H) Violin plot shows the expression level of Cck gene in all IT neurons at different postnatal stages. (TIF) [file pbio.3003594.s002.tif]

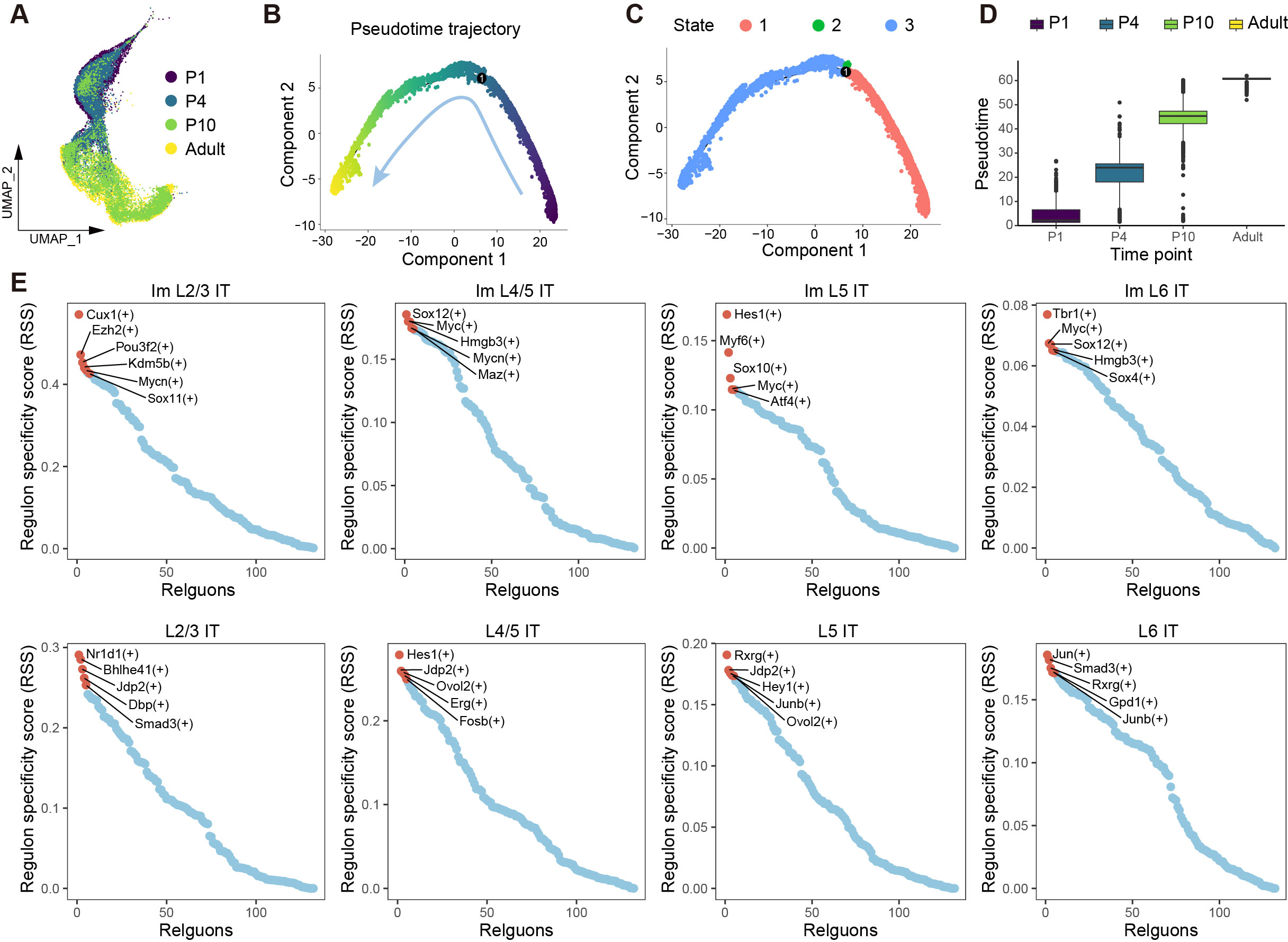

Supplement: S3 Fig — (A) Uniform Manifold Approximation Projection (UMAP) of all IT neurons in mouse PFC from scRNA-seq, which is colored by time stages. (B) Pseudotime trajectory of IT neurons, which is colored by pseudotime value. (C) Pseudotime trajectory of IT neurons, which is colored by pseudotime states. (D) Box plot shows the pseudotime distribution in each time stages. The data underlying this Figure can be found in S1 Data. (E) Regulon specificity score (RSS) ranking of relguons in each IT neuron subtype. (TIF) [file pbio.3003594.s003.tif]

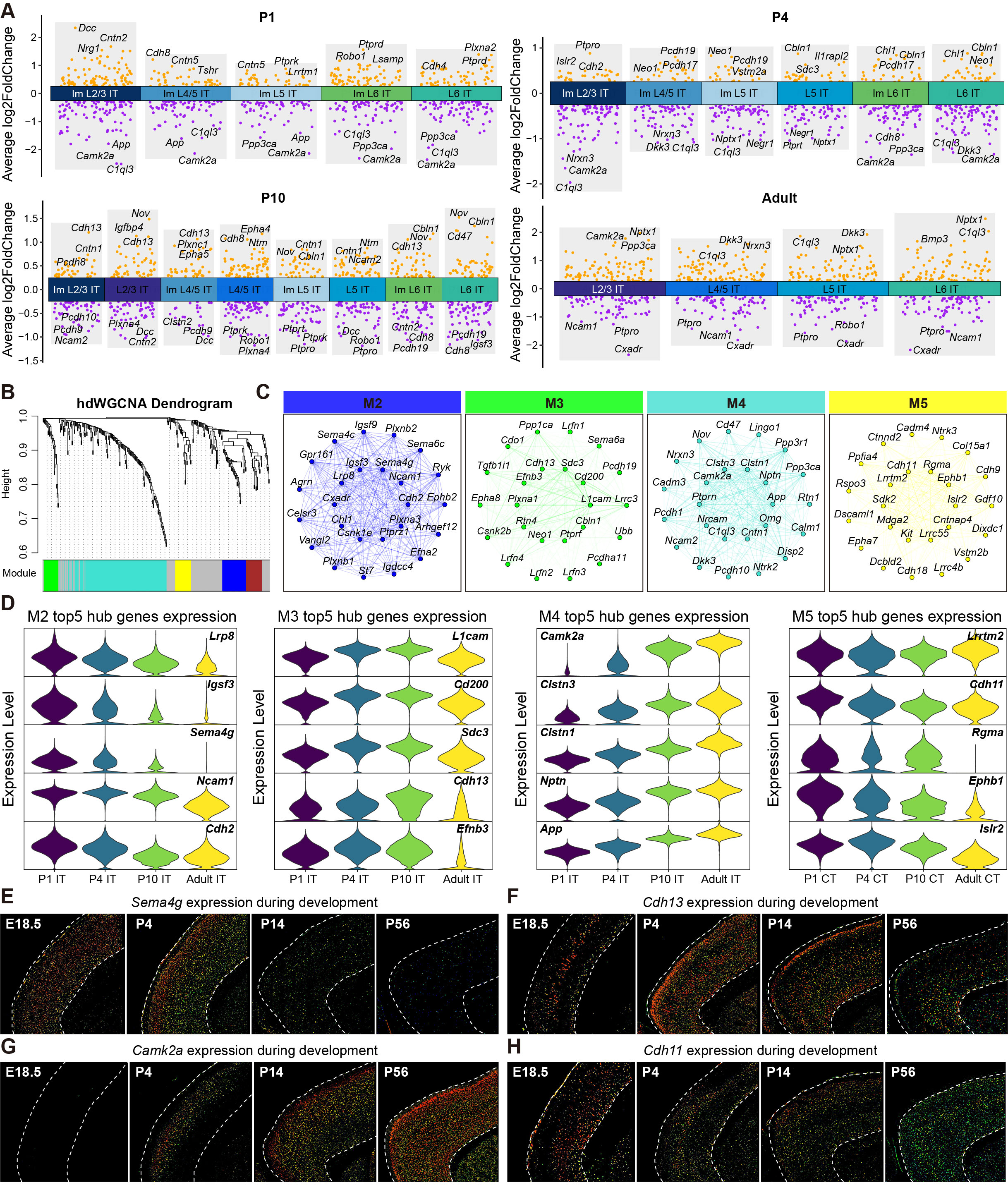

Supplement: S4 Fig — (A) Volcano plots show the differentially expressed cadherin and axon guidance genes of each IT neuron subtype at different developmental stages. For each stage, each IT neuron subtype is compared with other IT subtypes. Orange, up-regulated genes; purple, down-regulated genes. (B) hdWGCNA dendrogram of the co-expression network of cadherin and axon guidance genes. (C) Co-expression network diagram for module 2 to module 5. (D) Violin plots show the expression level of top 5 hub genes for module 2 to module 5 at different postnatal stages. (E–H) Allen mouse brain ISH images of Sema4g (E), Cdh13 (F), Camk2a (G), Cdh11 (H) gene at E18.5, P4, P14, and P56. (TIF) [file pbio.3003594.s004.tif]

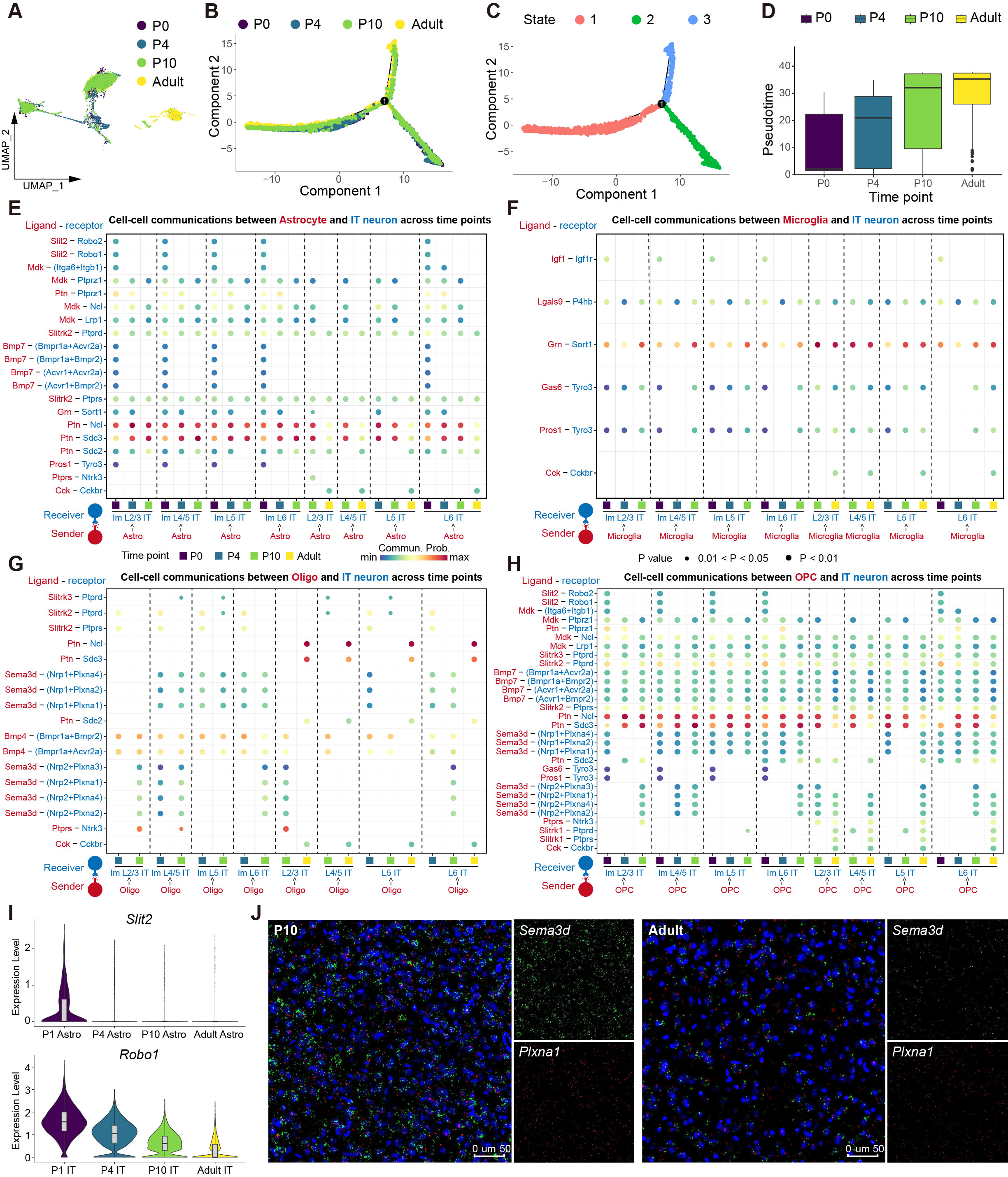

Supplement: S5 Fig — (A) Uniform Manifold Approximation Projection (UMAP) of all glial cells in mouse PFC from scRNA-seq, which is colored by time stages. Microglia are excluded due to different developmental origins. (B) Pseudotime trajectory of glial cells, which is colored by time stages. (C) Pseudotime trajectory of glial cells, which is colored by pseudotime states. (D) Box plot shows the pseudotime distribution in each time stages. The data underlying this Figure can be found in S1 Data. (E–H) Dot plots show significant ligand-receptor interactions among Astrocyte (E), Microglia (F), Oligo (G), OPC (H), and IT subtypes across time points. The dot color indicates the communication probability, and the dot size reflects the P value. The square color indicates time points. (I) Violin plots show the expression levels of the ligand gene Slit2 in astrocytes (top) and the receptor gene Robo1 in IT neurons (bottom) at different postnatal stages. (J) RNA FISH of Sema3d and Plxna1 ligand-receptor genes at P10 (left) and Adult (right) in mice PFC. The small boxes indicate the expression of each gene. Blue: DAPI, green: Sema3d, red: Plxna1. (TIF) [file pbio.3003594.s005.tif]

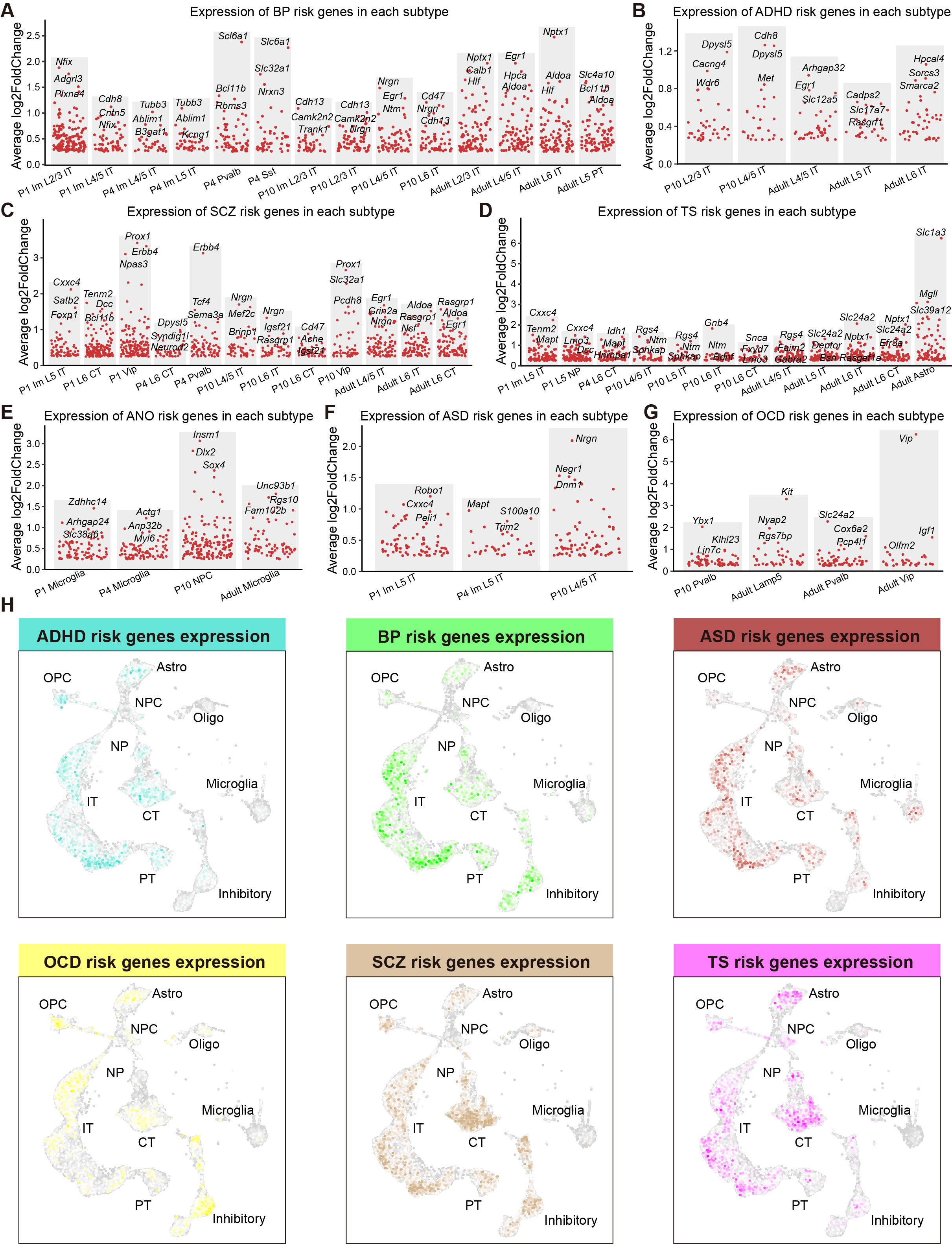

Supplement: S6 Fig — (A–G) Volcano plots show the differential expression of BP (A), ADHD (B), SCZ (C), TS (D), ANO (E), ASD (F), and OCD (G) risk genes across different postnatal stages in subtypes marked with asterisks in Fig 6A. The top 3 risk genes are displayed in each subtype. (H) Uniform Manifold Approximation Projection (UMAP) visualization of the ADHD, BP, ASD, OCD, SCZ, and TS scDRS disease score. (TIF) [file pbio.3003594.s006.tif]

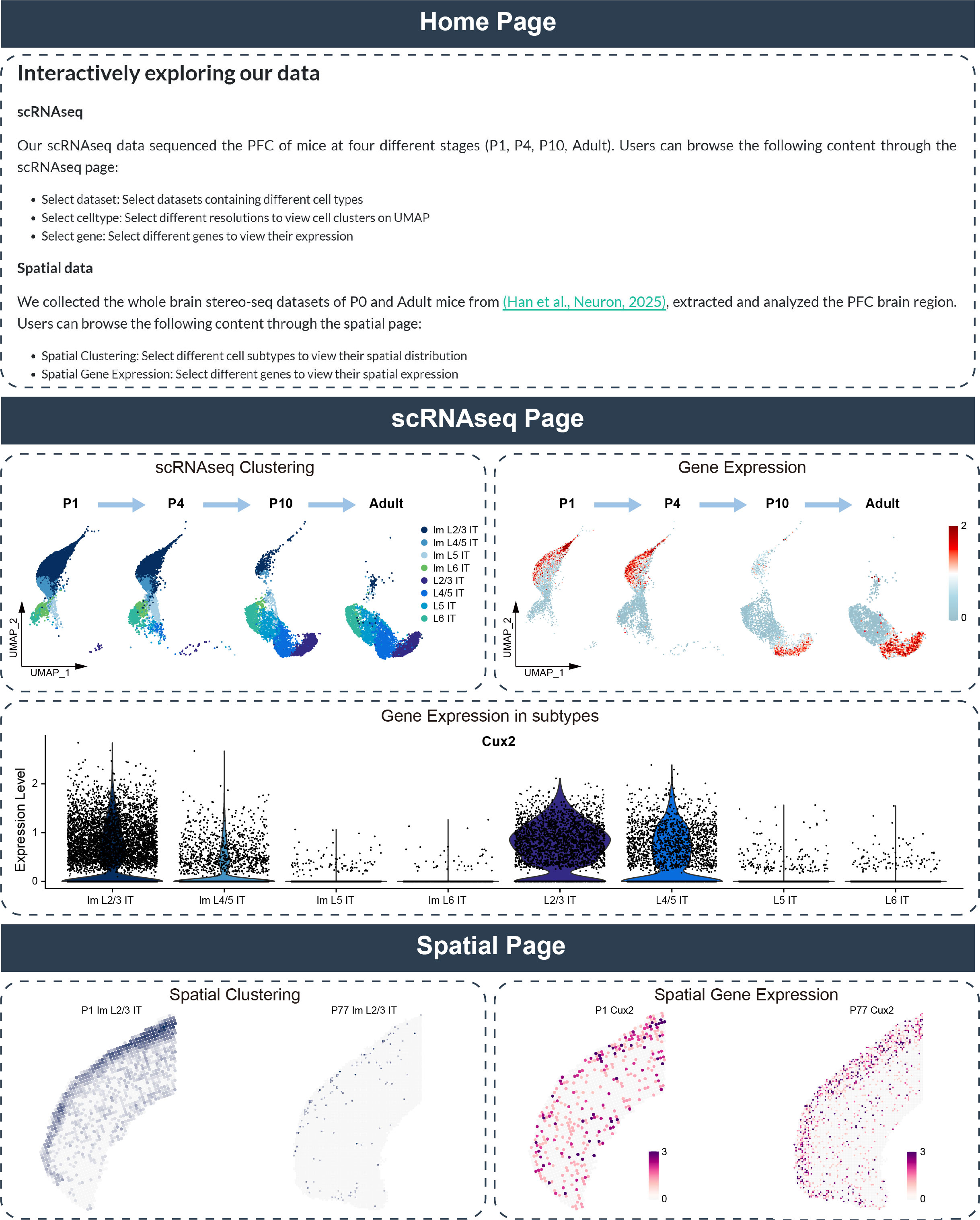

Supplement: S7 Fig — PFCdev-web is a shisny application that allows users to interactively access our data. Home Page provides information about our project and how to use it interactively. Users can access our scRNAseq data through scRNAseq Page, and access processed stereo-seq data through Spatial Page. (TIF) [file pbio.3003594.s007.tif]
