# Supplementary material for: A single-cell spatiotemporal transcriptomic atlas of mouse prefrontal cortex maps dynamics of intratelencephalic neurons during postnatal development
Source: PLoS Biol. 2026 Jan 12;24(1):e3003594. doi: 10.1371/journal.pbio.3003594 (PMC12818735; doi:10.1371/journal.pbio.3003594)
Supplement: Attachment — Submitted filename: Response to Reviewers.docx [file pbio.3003594.s020.docx]

**Editor’s comments:**

As you will see, the reviewers expressed interest in the resource value of the study as well as some of the findings. However, they also raised concerns about several aspects, including methodological concerns, missing information, and limitations in the comprehensiveness of the dataset. After discussing these points with the Academic Editor, we think that you should revise your study to address all of the methodological issues and requested clarifications, and provide a thorough response to all of the reviewers' points.

**Response:** We thank the editors and reviewers for the comments and feedback. We have revised our manuscript according to the reviewers' comments and provided a point-by-point response to each of the reviewers’ comments below. In our revised manuscript, we supplemented the information about all methodological issues and the missing information, and clarified the limitations of the comprehensiveness of the dataset. Meanwhile, we addressed the issues in the technical checks to meet the journal requirements. All changes have been marked with yellow highlight in our revised manuscript.

**Reviewer #1:**

In this manuscript, Zheng, Yan, and colleagues present a temporal, single-cell analysis of mouse prefrontal cortex during postnatal development. They show that intratelencephalic neurons exhibit the greatest transcriptomic diversity across postnatal timepoints and characterize molecular and cellular developmental patterns. While the manuscript offers a descriptive and useful resource for mouse prefrontal cortex postnatal development on a single-cell level, it presents few biological insights and most importantly provides insufficient methodological detail.

**Response:** We thank the reviewer for positive comments and valuable suggestions. We have made revisions according to the reviewers’ suggestions, including the issues on the biological insights and methodological detail. All changes have been marked with yellow highlight in our revised manuscript.

For the biological insights, in this study, we systematically dissected the dynamic changes in cell types and transcriptional profile during postnatal development of the mouse prefrontal cortex (PFC) by integrating single-cell RNA sequencing data and spatial transcriptomic data. Based on these comprehensive spatiotemporal atlases of PFC, we demonstrated that intratelencephalic (IT) neurons exhibit the most dramatic alterations during postnatal development, and identify the spatiotemporal dynamics of their differential gene expression.

In light of this, we reconstructed the developmental trajectory of PFC IT neurons and conducted in-depth investigations into the dynamic expression patterns of the key transcription factors and circuit wiring molecules. Our data revealed that these molecules exhibit distinct spatiotemporal expression patterns, which may play crucial roles in neuronal development and circuit formation.

In addition, we also found that glial cells play crucial roles in the development of neurons through dynamic cell-cell communication during postnatal PFC development. These spatiotemporal interactions are mediated by specific signaling molecules secreted by specific glial cells at different stages, which elegantly regulate the developmental processes of neurons such as synapse formation and myelination.

Importantly, our data revealed that neurological disease risk genes exhibit distinct cell-type-specific and developmental-stage-specific expression profiles. This discovery provided critical insights for understanding the pathogenesis of neurological diseases, suggesting that genetic susceptibility may contribute to disease onset by affecting specific cell subtypes during distinct developmental stages.

We have already discussed the biological insights in the Discussion section of our manuscript, and have also clarified the limitations of this study in the revised manuscript, in **Lines 466-473**.

For the missing methodological details, we have supplemented them to the Method section of the revised manuscript. Please see our point-by-point response in the following.

**Major concerns:**

- The number and sex of animals used in the experiment is not explicitly stated anywhere. How many mice are included in each age group?

**Response:** We apologize for overlooking this point. All mice used for single-cell RNA sequencing were female. Two mice at each time point were used for single-cell sequencing. To further validate our data, we also included spatial transcriptome data (Stereo-seq) (Han et al., 2025) and in situ hybridization (ISH) (Thompson et al., 2014) data from independent mice form public databases to confirm the single-cell data. The information of the experimental mice in these data was described in previous literature.

We have added this information to the **Methods** section of our revised manuscript, in **Lines 482-483** and **661-662**.

- The manuscript is missing information about several key methods:

1. The supervised classification framework used to identify temporal associations among neuronal subtypes.

**Response:** Thanks for the comment. To identify the associations among neuronal subtypes at different ages, we used XGBoost (Chen and Guestrin, 2016), a gradient boosted decision tree-based supervised classification framework. Three time periods, P1-P4, P4-P10, and P10-Adult were compared. 2000 highly variable genes were used as input features for XGBoost, and neuronal subtype labels were used as output. We trained XGBoost classifier to learn neuronal subtype labels from the previous age dataset, and used it to classify cells in the next age dataset. The correspondences between the true label of the next age dataset and the label assigned by the XGBoost classifier was used to map neuronal subtypes between different ages. The main steps are as follows: We use the R package xgboost (v1.7.5.1) to implement XGBoost supervised classification. The “xgb.DMatrix” function was used to construct the xgb.DMatrix object using 2000 highly variable genes and neuron subtype labels. We used the “xgb.cv” function to perform cross-validation to determine the optimal number of iterations. Then, the XGBoost model was trained using the “xgboost” function. Finally, the “predict” function was used to perform prediction.

As suggested, we have added this information to the **Methods** section of our revised manuscript, in **Lines 573-585**.

2. The method used for differential gene expression analysis and the threshold for significance.

**Response:** To perform differential gene expression analysis, we used the Wilcoxon rank-sum test, a nonparametric test, to compare whether there is a significant difference in the medians of two independent samples. Log2 fold change > 0.25 was used as the threshold to determine significant genes. Specifically, we used the “FindAllMarkers” function from the R package Seurat (Hao et al., 2021) (v4.4.0) to perform differential gene expression analysis with parameters: logfc.threshold = 0.25, test.use = "wilcox".

As suggested, we have added this information to the Methods section of our revised manuscript, in **Lines 586-591**.

3. The method for GO term analysis and the threshold for significance.

**Response:** GO terms enrichment analysis was performed using gProfiler (Raudvere et al., 2019) with default parameters. The adjusted p-values < 0.05 was used as the significance threshold. And the results of GO terms were limited to enrichment in GO biological processes.

As suggested, we have added this information to the Methods section of our revised manuscript, in **Lines 592-595**.

4. Differential gene expression analysis of genes along pseudotime. How was this done? Were the authors comparing differences in gene expression between pseudotime states? This was unclear.

**Response:** The differential gene expression analysis of genes along pseudotime was performed using the Generalized Additive Model (GAM) in Monocle2 (Qiu et al., 2017) (v2.24.0). Monocle2 assigned each cell a "pseudotime" value, which recorded its progress through the process in the experiment. The GAM can model a gene's expression level as a smooth, nonlinear function of pseudotime. Then, likelihood ratio test (LRT) was used to identify pseudotime related genes. The main steps are as follows: We used the “differentialGeneTest” function from the R package Monocle2 (v2.24.0) to find differentially expressed genes related to pseudotime with parameter: fullModelFormulaStr = "~sm.ns(Pseudotime)". The q-values < 0.01 was used as the significance threshold.

As suggested, we have added this information to the Methods section of our revised manuscript, in **Lines 604-612**.

5. Clustering of pseudotime DEGs into modules. How were the genes clustered/how were these modules identified?

**Response:** We used "ward.D2", a hierarchical clustering algorithm, to cluster pseudotime DEGs into modules. Specifically, we used the “plot_pseudotime_heatmap” function from the R package Monocle2 (v2.24.0) to plot a heatmap of differentially expressed genes along pseudotime with parameter: hclust_method = "ward.D2".

As suggested, we have added this information to the Methods section of our revised manuscript, in **Lines 613-616**.

- The GWAS risk genes used in this analysis do not fully reflect the current state of knowledge on psychiatric disorders. Given that these are highly polygenic traits with many small-effect variants contributing, it would be more informative to use a method, such as single-cell Disease Relevance Score (scDRS), that uses the latest GWAS summary statistics to provide a more comprehensive view of how polygenic risk maps onto the cell types and developmental stages.

**Response:** We appreciate the reviewers' suggestions. We have reanalyzed psychiatric disorders using scDRS and the GWAS summary statistics. The reanalyzed figure have been updated to **Fig. 6** and **Fig. S6**. The information has been updated in the revised manuscript, in **Lines 369-413** of the Result section, **Lines 453-458** of the Discussion section, and **Lines 649-657** of the Method section.

The neurological diseases GWAS summary statistics were collected from previous studies (**Table S7**) (Arnold et al., 2018; Demontis et al., 2019; Duncan et al., 2017; Grove et al., 2019; Howard et al., 2019; Lee et al., 2019; Mullins et al., 2021; Pardiñas et al., 2018; Yu et al., 2019). First, we used MAGMA (v1.10) (de Leeuw et al., 2015) to perform SNP annotation and GWAS disease risk gene weighting. The top 1000 GWAS disease risk genes by weight score were used for further analysis (**Table S8**). Then we used scDRS (v1.0.4) (Zhang et al., 2022) to perform disease risk genes enrichment analysis. Briefly, “scdrs compute-score” command was used to evaluate disease enrichment to individual cells. Then, “scdrs perform-downstream” command was used to obtain the group-level statistics for each cell type. All parameters are set to the default values according to scDRS documentation. The P-value < 0.05 was used as the threshold of significance.

We investigated the enrichment of risk genes for eight neurological disorders in diverse PFC cell subtypes during postnatal development. These diseases include attention deficit hyperactivity disorder (ADHD), anorexia nervosa (ANO), autism spectrum disorder (ASD), bipolar disorder (BP), major depressive disorder (MDD), obsessive-compulsive disorder (OCD), schizophrenia (SCZ), and tourette syndrome (TS). Overall, risk genes associated with different diseases tend to be enriched in specific cell subtypes of mouse PFC (**Response Fig. 1A**). For example, risk genes linked to ADHD, ASD, BP, SCZ and TS are primarily enriched in excitatory neuronal subtypes, while risk genes associated with OCD are mainly enriched in inhibitory neuronal subtypes. And risk genes associated with MDD are enriched in both excitatory and inhibitory neuronal subtypes. ANO related risk genes are mainly enriched in microglia subtype (**Response Fig. 1A, C and Response Fig. 2H**). These findings emphasize the cell type specificity of neurological disease risk, suggesting that genetic susceptibility may converge on certain cell subtypes during postnatal stages.

We further analyzed the temporal expression patterns of disease-associated risk genes in each cell subtype enriched for different neurological diseases. The top 1000 GWAS disease risk genes by weight score were used for analysis (**Table S8**). Differential gene expression analysis suggested that these disease risk genes may function at different developmental ages and specific cell subtypes (**Response Fig. 1B and Response Fig. 2A-G**). For example, MDD risk genes were primarily enriched in neuronal subtypes, which agreed with previous study indicating that alterations in gene expression of glutamatergic and GABAergic neurons may lead to MDD symptoms (Sequeira et al., 2009). ANO risk genes were enriched in microglia subtype, showing distinct enrichment patterns (**Response Fig. 1C, D**). Previous studies have reported a reduction in the number of microglia in ANO mice (Verspohl et al., 2025). *Tcf4* and *Egr1* are two risk genes for MDD, and their expression is reduced in MDD patients according to previous studies (Bristot et al., 2024; Mossakowska-Wójcik et al., 2018). They show different temporal expression patterns. Our data indicated that *Tcf4* in IT neurons was mainly expressed at P1, while *Egr1* was predominantly expressed at adult (**Response Fig. 1D, E**). Allen ISH results further validated the expression patterns of these genes across different postnatal ages (**Response Fig. 1F**). Together, these results depict how risk genes for various neurological diseases are enriched in specific cell subtypes at different postnatal time points, helping to understand the causes of diseases and discover therapeutic targets.

**Response Fig.1.** **Enrichment analysis of neurological developmental disease risk genes in diverse cell subtype of mouse PFC during postnatal development**

(A) Barplot shows the enrichment levels of GWAS candidate genes from 8 PFC-related disease in different cell subtypes. The bar heights represent mean disease score calculated by scDRS, annotated with time stages and cell subtypes. Asterisks denote the cell subtypes with P-value < 0.05. ADHD: attention deficit hyperactivity disorder; ANO: anorexia nervosa; ASD: autism spectrum disorder; BP: bipolar disorder; MDD: major depressive disorder; OCD: obsessive-compulsive disorder; SCZ: schizophrenia; TS: tourette syndrome.

(B) Volcano plot shows the differential expression of MDD risk genes across different postnatal stages in subtypes marked with asterisks in (A). The top 3 risk genes are displayed in each subtype.

(C) UMAP visualization of the MDD (left) and ANO (right) scDRS disease score.

(D) Heatmap shows the expression of MDD and ANO risk genes in their enriched subtypes.

(E) Violin plot shows the expression level of *Tcf4* (left) and *Egr1* (right) genes in MDD enriched IT neurons at different ages.

(F) Allen mouse brain ISH images of *Tcf4* (up) and *Egr1* (bottom) genes at E18.5, P4, P14 and P56.

**Response Fig.2.** **Expression of neurological disease risk genes in mouse PFC**

(A-G) Volcano plots show the differential expression of BP (A), ADHD (B), SCZ (C), TS (D), ANO (E), ASD (F), and OCD (G) risk genes across different postnatal stages in subtypes marked with asterisks in Fig 6A. The top 3 risk genes are displayed in each subtype.

(H) UMAP visualization of the ADHD, BP, ASD, OCD, SCZ and TS scDRS disease score.

**Minor concerns:**

- The authors report significant changes in the proportions of IT neurons between developmental stages but have not quantified this. A statistical method that can account for variability between samples (e.g., propeller method) is needed to make this claim.

**Response:** We appreciate the reviewers' suggestions. We used the coefficient of variation, a common statistical method for measuring the degree of variability in samples, to quantify the changes in the proportion of different neuronal subtypes between developmental stages. As shown in **Response Fig. 3**, quantitative analysis showed that the IT neuronal subtypes exhibited more significant changes compared to other neuronal subtypes.

As suggested, we have added this figure to **Fig. S1E** and updated the information of our revised manuscript, in **Lines 112-113** and **973-974**.

**Response Fig.3.** **Quantification of changes in the proportion of neuron subtypes during development**

Bar plot shows the coefficient of variation of the changes in the proportion of different neuronal subtypes during development.

- It would be helpful to include a full section from the Allen Mouse Brain ISH atlas as reference to indicate where the regions of interest are located.

**Response:** We appreciate the reviewers' suggestions. As shown in **Response Fig. 4**, a full sagittal section of Allen Mouse Brain atlas is displayed, with the PFC region circled in red color.

As suggested, we have added this figure to **Fig. S1F** and updated the information of our revised manuscript, in **Lines 120** and **975**.

**Response Fig.4.** **Allen Mouse Brain atlas**

A full sagittal section of Allen Mouse Brain atlas with the PFC region circled in red color.

- The circle volcano plots in Figure 2D and 4A are difficult to interpret without axes. I recommended replacing these figures with plots like those in Fig S2D and Fig S4A.

**Response:** We appreciate the reviewers' suggestions. We have replaced the circle volcano plots in **Fig. 2D and 4A** with horizontal volcano plots and updated the information of revised manuscript, in **Lines 151** and **259**.

- It is unclear why mature some L6 IT neurons appear early in pseudotime and some NPCs are positioned later in pseudotime. The authors should clarify this apparent discrepancy.

**Response:** Based on previous reports and our data, neurons develop in the order of deep-layer first and then superficial-layer (Cheng et al., 2022; Klingler et al., 2021). Some L6 IT neurons have already matured in the early ages (P1 and P4). And pseudotime is closely related to developmental age, so some mature L6 IT neurons appear early in the pseudotime axis. Some NPCs positioned later in the pseudotime axis is because NPCs still exist in the P10 age, reflecting the plasticity of neurons in the first two weeks after birth (Espinosa and Stryker, 2012; Innocenti and Price, 2005).

We have clarified them in the Result section of our revised manuscript (“We observed some L6 IT neurons appear early in pseudotime. This is because deep neurons develop first, so some L6 IT neurons have already matured at P1.” in **Lines 195-196** and “Some NPCs positioned later in the pseudotime axis is because NPCs still exist in the P10 age, reflecting the plasticity of neurons in the first two weeks after birth.” in **Lines 311-313**).

- Please randomize the plotting order of points in the pseudotime trajectory in Fig S3B so that all ages are clearly visible. Once corrected, this figure would be more informative than Fig 3B and the authors could consider using it as a replacement for Fig 3B.

**Response:** We appreciate the reviewers' suggestions. We have randomized the plotting order of points in the pseudotime trajectory in **Fig. S3B** and replaced **Fig. 3B** and updated the information of revised manuscript, in **Lines 207** and **995**.

- It is unclear whether the analysis in Figure 4B is comparing between ages or IT neuron subtypes. If it is the latter, the authors should discuss why several of the same genes (e.g., Cbln1, Chl1, Nov, Cdh13, Cntn1) are shared across several subtypes.

**Response:** We apologize for the unclear description and have corrected it in the revised manuscript in **Lines 250-251**.

We compared the differentially expressed cadherins and axon guidance genes between IT neuronal subtypes. **Fig. 4B** shows the top2 cadherin and axon guidance genes in each IT subtype. The complete differential expression analysis results are shown in the volcano plot of **Fig. S4A**. Although we selected significant genes through differential analysis, this does not mean that these genes are expressed only in a single subtype. In fact, many cadherins and axon guidance genes are expressed across multiple layers. For example, *Cbln1* and *Chl1* are mainly expressed in the deep-layers L5 and L6 at P4, while the *Cdh13* gene is mainly expressed in the superficial-layers L2/3 and L4/5 at P10.

- Are the genes in Module 1 already expressed in deep-layer IT neurons earlier in development (e.g., E18.5)? This would help clarify whether M1 is truly subtype-specific to L2/3 IT neurons or whether it reflects a maturation program that first appears in deeper layers and then becomes enriched in more superficial layers between P1-P4.

**Response:** We appreciate the reviewers' suggestions. As shown in **Response Fig. 5**, Allen ISH results indicated that the gene *Cntn2* in Module 1 was already expressed in deep layers before birth (E15.5) and gradually enriched in superficial layers after birth. This indeed reflects a maturation program that first appears in deeper layers and then becomes enriched in more superficial layers.

Therefore, the description "subtype-specific" is inaccurate, and we have changed it to "subtype enrichment" in the revised manuscript in **Line 279**.

**Response Fig.5.** **Allen mouse brain ISH images of *Cntn2* gene at E15.5, E18.5, P4, P14 and P56.**

The figure shows that the gene *Cntn2* in Module 1 is enriched in the deeper layers at E15.5, and gradually becomes enriched in superficial layers during development.

- Fig 5K is missing a legend. Please add this to help with interpretation.

**Response:** Based on the reviewer2’s major comment3, we have removed this plot and replaced it with **Fig. 5J** in the revised manuscript.

**Reviewer #2:**

This study combined scRNA-seq with spatial transcriptomics across multiple developmental stages (P1, P4, P10, P84) to provide a high-resolution spatiotemporal map of neuronal and glial maturation in mouse PFC. The study advances understanding of excitatory neuron subtype development, transcription factor networks, axon guidance programs, and neuron-glia signaling. Addtionally, the study provided enrichment analysis of GWAS risk genes (ASD, SCZ, MDD, etc.) across cell types and developmental windows provides translational relevance, linking developmental PFC molecular programs to human disease risk. However, several concerns reduced the significance of this study.

**Response:** We thank the reviewer for positive comments and valuable suggestions. We have performed the experiments and carefully revised our manuscript according to the reviewers’ suggestions. All changes have been marked with yellow highlight in our revised manuscript. Please see our point-by-point response in the following.

**Major concerns:**

1. The study is largely descriptive and with limited mechanistic insight. While transcriptomic and computational analyses are strong, experimental validation (e.g., perturbation of key TFs or signaling pathways) is lacking.

**Response:** We agree with the reviewer that further experimental validation will provide more mechanical insight. However, as a "Methods & Resources" type article, this study aims to provide and mine useful data. Nevertheless, for key genes such as TFs, cadherin, and axon guidance genes, we still provided the in situ validation images from Allen ISH, such as **Fig. 1E**, **Fig. 2G-J**, **Fig. 3H-K**, **Fig. 4G**, **Fig. 6F**, **Fig. S1D**, **Fig. S4E-H**. In addition, we have performed RNA FISH assay to support the co-localization validation of ligand-receptor genes, as shown in **Response Fig.6**. Although we did not perform perturbation experiment for key TFs or signaling pathways, previous studies were able to verify some of the genes we analyzed. For example, our data show that the *Sox11* TFs is mainly expressed in immature L2/3 IT subtype, which is consistent with previous reports that overexpression of *Sox11* affects the circuit development of L2/3 IT neurons in mouse motor cortex (Klingler et al., 2021). And our data show that astrocytes at P1 activate the SLIT signaling pathway, which is consistent with previous reports that slit-robo signaling is associated with axon repulsion function during early nervous system development (Blockus and Chédotal, 2016).

We acknowledge these limitations and have clarified them in the Discussion section of our revised manuscript (“And the functions of some key TFs, circuitry molecules and signaling pathways also require further experimental investigation.” in **Lines 467-468**).

2. Four time points (P1, P4, P10, P84) were used, but the spatial data is missing (P1 and P77 are re-analyzed). This should be clearly stated and with own spatial data generated to fill in the time points. In addition, ideally, intermediate stages (e.g., P14, P21, adolescence) would provide a more continuous trajectory of PFC maturation.

**Response:** We agree with the reviewer that more data would make this study more comprehensive. However, this would significantly increase the cost and have relatively limited impact on the results of the study. The purpose of re-analyzing the spatial data is primarily to further validate the results from our single-cell data. The P1 and P77 spatial data are sufficient to support our main findings: IT neurons in the early postnatal mouse PFC are mainly immature IT neurons, while IT neurons in the adult mouse PFC are mainly mature IT neurons. Although spatial data for P4 and P10 are missing, we have supplemented the data with Allen ISH in situ hybridization results at four time points (E18.5, P4, P14, P56), as shown in **Fig. 1E** and **Fig. S1D**. In fact, current spatial transcriptomics technology still has many shortcomings, such as low efficiency in capturing single gene (You et al., 2024). Therefore, we only used it in Figure 1 to map the spatial distribution of cell subtypes. For spatial validation of individual genes, we used Allen ISH results, which have higher capture efficiency.

Ideally, adding data from intermediate stages (such as P14, P21, and adolescence) would provide a more continuous trajectory of PFC maturation. However, in this study, we primarily focused on PFC development in mice during the first two postnatal weeks. According to previous reports, the first two postnatal weeks constitute a crucial period for IT axon growth and circuit formation: axons typically reach their target regions within the first week and undergo subsequent refinement through activity-dependent pruning (Chini and Hanganu-Opatz, 2021; Lewis et al., 2013). Furthermore, as shown in **Fig. 1F**, **Fig. 2B** and **Fig. 3A**, our data indicate that 83% of the IT neurons have already developed into mature IT neurons at P10. Therefore, we believe that at P14, P21, and adolescence stages, IT subtypes tend to be less dynamic and have relatively limited impact on the maturation trajectory.

We acknowledge these limitations and have clarified them in the Discussion section of our revised manuscript (“However, further research with more age stages single-cell and spatial transcriptome data is needed for a more comprehensive understanding of PFC maturation.” in **Lines 466-467**).

3. CellChat predictions are insightful, but direct evidence (e.g., ligand-receptor validation, co-localization, or functional manipulation) is missing. Additional experimental results are required to support the conclusions.

**Response:** We appreciate the reviewers' suggestions. As shown in **Response Fig. 6A**, we validated the co-localization expression of the ligand-receptor genes *Sema3d* and *Plxna1* using RNA FISH assay. To quantify gene expression, we segmented cells using cellpose (Stringer et al., 2021) and then calculated the fluorescence intensity of *Sema3d* and *Plxna1* in each cell as the expression level. Then, we used SpaGene (Budhkar et al., 2025) software to calculate the strongest ligand-receptor interaction between each cell and its five spatial nearest neighbors as the ligand-receptor co-localization expression level. As shown in **Response Fig. 6B-C**, *Sema3d* and *Plxna1* show significantly stronger co-localization expression at P10 compared to Adult, consistent with our single-cell sequencing analysis results (**Fig. 5I**).

Although we did not perform functional manipulation experiment, previous studies were able to verify some of the ligand-receptor genes we analyzed. For example, our data show that astrocytes at P1 activate the SLIT signaling pathway, which is consistent with previous reports that slit-robo signaling is associated with axon repulsion function during early nervous system development (Blockus and Chédotal, 2016). And, our data show that oligodendrocytes at P4 and P10 activate the SEMA3 signaling pathway, while the *Sema3d* gene is associated with early axonal fasciculation in neurons (Wolman et al., 2007).

Based on the reviewer’s recommendation, we have added this figure to **Fig. 5J-K** and **Fig. S5J** to replace the previous results and updated the information of revised manuscript, in **Lines 336-338, 357-359, 508-533, 640-648, 1022-1023**. And we have added a discussion about the ligand-receptor functionality in the Discussion section of our revised manuscript (“Our intercellular communication analysis revealed several important signaling pathways (such as SLIT and SEMA3) may mediate glia-IT neurons interaction, which would play important roles in axon guidance and fasciculation of PFC IT neurons” in **Lines 442-444**).

**Response Fig.6.** **ligand-receptor validation and co-localization analysis.**

(A) RNA FISH of *Sema3d* and *Plxna1* ligand-receptor genes at P10 (left) and Adult (right) in mice PFC. The small boxes indicate the expression of each gene. Blue: DAPI, green: *Sema3d*, red: *Plxna1*.

(B) Visualization of co-localization expression of *Sema3d* and *Plxna1* in P10 (left) and Adult (right) mice PFC after cell segmentation. Color indicates the intensity of co-localization expression.

(C) Quantitative comparison of *Sema3d* and *Plxna1* co-localization expression in P10 and Adult mice PFC.

**Minor concerns:**

1. Other populations (e.g., PT, CT, interneurons) are noted as stable, but their contributions to PFC development and disease are under-discussed.

**Response:** Thanks for the comment. Our data showed that IT neurons exhibited the greatest transcriptomic diversity during PFC postnatal development, while PT, CT, and interneurons showed relatively stable transcriptomic profiles (**Fig. 2B-C**). Therefore, our study primarily focused on the development of IT neurons. According to previous study on the mouse cortex, PT, CT, and interneurons developed relatively earlier and have already matured prenatally (Di Bella et al., 2021). Abnormal development of any of these neuronal subtypes may lead to neurological diseases. IT/PT imbalance is a cause of many neurological diseases such as autism, schizophrenia, and depression (Shepherd, 2013). Abnormalities in CT neural circuits may lead to diseases such as schizophrenia and bipolar disorder (Angulo Salavarria et al., 2023). Disruption of the development or function of GABAergic interneurons may lead to epilepsy and other neuropsychiatric disorders associated with cognitive impairment (Marín, 2012).

We have added a discussion about PT, CT and interneurons in the Discussion section of our revised manuscript (“Notably, other neuronal subtypes (e.g., L5 PT, L6 CT and inhibitory neurons) show relatively consistent transcriptomic profiles during postnatal development, consistent with the view that deep layer neurons mature first and superficial layer neurons mature later during cortical development.” in **Lines 424-427** and “For example, IT/PT imbalance is a cause of many neurological diseases such as autism, schizophrenia, and depression. Abnormalities in CT neural circuits may lead to diseases such as schizophrenia and bipolar disorder. Disruption of the development or function of GABAergic interneurons may lead to epilepsy and other neuropsychiatric disorders associated with cognitive impairment.” in **Lines 449-453**).

2. While code and data are deposited, more explicit instructions or tutorials for reanalysis would enhance utility for the broader community.

**Response:** We appreciate the reviewers' suggestions. We have provided more detailed instructions and tutorials for reanalysis on GitHub. Furthermore, to make it easier for users to explore our data, we have also developed a user-friendly online website, as shown in **Fig. S7**. Users can use the website to view our single-cell and spatial transcriptome data and explore the expression of genes they are interested in.

3. this study reference human studies, but cross-species relevance (especially timing differences between mouse and human PFC development) should be more explicitly addressed.

**Response:** We appreciate the reviewers' suggestions. Indeed, all analyses in this study were performed on mice. Although some studies have reported certain conservation of neocortical cell types across mammalian species (Chen et al., 2023; Han et al., 2025; Yuan et al., 2022), considering the timing differences in PFC development among different species, especially between mice and human, it remains unclear whether the cell types and gene expression dynamics during PFC development in mice are also applicable to other species.

We acknowledge these limitations and have clarified them in the Discussion section of our revised manuscript (“Additionally, all analyses in this study were performed on mice. Although some studies have reported certain conservation of neocortical cell types across mammalian species, considering the timing differences in PFC development among different species, especially between mice and human, it remains unclear whether the cell types and gene expression dynamics during PFC development in mice are also applicable to other species.” in **Lines 469-473**).

**References**

Angulo Salavarria, M.M., Dell'Amico, C., D'Agostino, A., Conti, L., and Onorati, M. (2023). Cortico-thalamic development and disease: From cells, to circuits, to schizophrenia. Frontiers in neuroanatomy *17*, 1130797.

Arnold, P.D., Askland, K.D., Barlassina, C., Bellodi, L., Bienvenu, O.J., Black, D., Bloch, M., Brentani, H., Burton, C.L., Camarena, B.*, et al.* (2018). Revealing the complex genetic architecture of obsessive–compulsive disorder using meta-analysis. Molecular psychiatry *23*, 1181-1188.

Blockus, H., and Chédotal, A. (2016). Slit-Robo signaling. Development (Cambridge, England) *143*, 3037-3044.

Bristot, G., Feiten, J.G., Pfaffenseller, B., Hizo, G.H., Possebon, G.M.P., Valiati, F.E., Pinto, J.V., Caldieraro, M.A., Fleck, M.P.A., Gama, C.S.*, et al.* (2024). Early growth response 1 (EGR1) is downregulated in peripheral blood from patients with major psychiatric disorders. Trends in psychiatry and psychotherapy *46*.

Budhkar, A., Ha, J., Song, Q., Su, J., and Zhang, X. (2025). SpaGene: A Deep Adversarial Framework for Spatial Gene Imputation. bioRxiv : the preprint server for biology.

Chen, A., Sun, Y., Lei, Y., Li, C., Liao, S., Meng, J., Bai, Y., Liu, Z., Liang, Z., Zhu, Z.*, et al.* (2023). Single-cell spatial transcriptome reveals cell-type organization in the macaque cortex. Cell *186*, 3726-3743.e3724.

Chen, T., and Guestrin, C. (2016). XGBoost: A Scalable Tree Boosting System. In Proceedings of the 22nd ACM SIGKDD International Conference on Knowledge Discovery and Data Mining (San Francisco, California, USA: Association for Computing Machinery), pp. 785–794.

Cheng, S., Butrus, S., Tan, L., Xu, R., Sagireddy, S., Trachtenberg, J.T., Shekhar, K., and Zipursky, S.L. (2022). Vision-dependent specification of cell types and function in the developing cortex. Cell *185*, 311-327.e324.

Chini, M., and Hanganu-Opatz, I.L. (2021). Prefrontal Cortex Development in Health and Disease: Lessons from Rodents and Humans. Trends in neurosciences *44*, 227-240.

de Leeuw, C.A., Mooij, J.M., Heskes, T., and Posthuma, D. (2015). MAGMA: generalized gene-set analysis of GWAS data. PLoS computational biology *11*, e1004219.

Demontis, D., Walters, R.K., Martin, J., Mattheisen, M., Als, T.D., Agerbo, E., Baldursson, G., Belliveau, R., Bybjerg-Grauholm, J., Bækvad-Hansen, M.*, et al.* (2019). Discovery of the first genome-wide significant risk loci for attention deficit/hyperactivity disorder. Nature genetics *51*, 63-75.

Di Bella, D.J., Habibi, E., Stickels, R.R., Scalia, G., Brown, J., Yadollahpour, P., Yang, S.M., Abbate, C., Biancalani, T., Macosko, E.Z.*, et al.* (2021). Molecular logic of cellular diversification in the mouse cerebral cortex. Nature *595*, 554-559.

Duncan, L., Yilmaz, Z., Gaspar, H., Walters, R., Goldstein, J., Anttila, V., Bulik-Sullivan, B., Ripke, S., Thornton, L., Hinney, A.*, et al.* (2017). Significant Locus and Metabolic Genetic Correlations Revealed in Genome-Wide Association Study of Anorexia Nervosa. The American journal of psychiatry *174*, 850-858.

Espinosa, J.S., and Stryker, M.P. (2012). Development and plasticity of the primary visual cortex. Neuron *75*, 230-249.

Grove, J., Ripke, S., Als, T.D., Mattheisen, M., Walters, R.K., Won, H., Pallesen, J., Agerbo, E., Andreassen, O.A., Anney, R.*, et al.* (2019). Identification of common genetic risk variants for autism spectrum disorder. Nature genetics *51*, 431-444.

Han, L., Liu, Z., Jing, Z., Liu, Y., Peng, Y., Chang, H., Lei, J., Wang, K., Xu, Y., Liu, W.*, et al.* (2025). Single-cell spatial transcriptomic atlas of the whole mouse brain. Neuron *113*, 2141-2160.e2149.

Hao, Y., Hao, S., Andersen-Nissen, E., Mauck, W.M., 3rd, Zheng, S., Butler, A., Lee, M.J., Wilk, A.J., Darby, C., Zager, M.*, et al.* (2021). Integrated analysis of multimodal single-cell data. Cell *184*, 3573-3587.e3529.

Howard, D.M., Adams, M.J., Clarke, T.K., Hafferty, J.D., Gibson, J., Shirali, M., Coleman, J.R.I., Hagenaars, S.P., Ward, J., Wigmore, E.M.*, et al.* (2019). Genome-wide meta-analysis of depression identifies 102 independent variants and highlights the importance of the prefrontal brain regions. Nature neuroscience *22*, 343-352.

Innocenti, G.M., and Price, D.J. (2005). Exuberance in the development of cortical networks. Nature reviews Neuroscience *6*, 955-965.

Klingler, E., Tomasello, U., Prados, J., Kebschull, J.M., Contestabile, A., Galiñanes, G.L., Fièvre, S., Santinha, A., Platt, R., Huber, D.*, et al.* (2021). Temporal controls over inter-areal cortical projection neuron fate diversity. Nature *599*, 453-457.

Lee, P.H., Anttila, V., Won, H., Feng, Y.-C.A., Rosenthal, J., Zhu, Z., Tucker-Drob, E.M., Nivard, M.G., Grotzinger, A.D., Posthuma, D.*, et al.* (2019). Genomic Relationships, Novel Loci, and Pleiotropic Mechanisms across Eight Psychiatric Disorders. Cell *179*, 1469-1482.e1411.

Lewis, T.L., Jr., Courchet, J., and Polleux, F. (2013). Cell biology in neuroscience: Cellular and molecular mechanisms underlying axon formation, growth, and branching. The Journal of cell biology *202*, 837-848.

Marín, O. (2012). Interneuron dysfunction in psychiatric disorders. Nature reviews Neuroscience *13*, 107-120.

Mossakowska-Wójcik, J., Orzechowska, A., Talarowska, M., Szemraj, J., and Gałecki, P. (2018). The importance of TCF4 gene in the etiology of recurrent depressive disorders. Progress in neuro-psychopharmacology & biological psychiatry *80*, 304-308.

Mullins, N., Forstner, A.J., O'Connell, K.S., Coombes, B., Coleman, J.R.I., Qiao, Z., Als, T.D., Bigdeli, T.B., Børte, S., Bryois, J.*, et al.* (2021). Genome-wide association study of more than 40,000 bipolar disorder cases provides new insights into the underlying biology. Nature genetics *53*, 817-829.

Pardiñas, A.F., Holmans, P., Pocklington, A.J., Escott-Price, V., Ripke, S., Carrera, N., Legge, S.E., Bishop, S., Cameron, D., Hamshere, M.L.*, et al.* (2018). Common schizophrenia alleles are enriched in mutation-intolerant genes and in regions under strong background selection. Nature genetics *50*, 381-389.

Qiu, X., Mao, Q., Tang, Y., Wang, L., Chawla, R., Pliner, H.A., and Trapnell, C. (2017). Reversed graph embedding resolves complex single-cell trajectories. Nature methods *14*, 979-982.

Raudvere, U., Kolberg, L., Kuzmin, I., Arak, T., Adler, P., Peterson, H., and Vilo, J. (2019). g:Profiler: a web server for functional enrichment analysis and conversions of gene lists (2019 update). Nucleic acids research *47*, W191-w198.

Sequeira, A., Mamdani, F., Ernst, C., Vawter, M.P., Bunney, W.E., Lebel, V., Rehal, S., Klempan, T., Gratton, A., Benkelfat, C.*, et al.* (2009). Global brain gene expression analysis links glutamatergic and GABAergic alterations to suicide and major depression. PloS one *4*, e6585.

Shepherd, G.M. (2013). Corticostriatal connectivity and its role in disease. Nature reviews Neuroscience *14*, 278-291.

Stringer, C., Wang, T., Michaelos, M., and Pachitariu, M. (2021). Cellpose: a generalist algorithm for cellular segmentation. Nature methods *18*, 100-106.

Thompson, Carol L., Ng, L., Menon, V., Martinez, S., Lee, C.-K., Glattfelder, K., Sunkin, Susan M., Henry, A., Lau, C., Dang, C.*, et al.* (2014). A High-Resolution Spatiotemporal Atlas of Gene Expression of the Developing Mouse Brain. Neuron *83*, 309-323.

Verspohl, V., van Egmond, M., Kneisel, L., Reese, F., Thelen, A.C., Korten, N., Neumann, M., Schaack, L., Voelz, C., Käver, L.*, et al.* (2025). Chronic starvation induces microglial cell depletion in an activity-based anorexia model. Scientific reports *15*, 14132.

Wolman, M.A., Regnery, A.M., Becker, T., Becker, C.G., and Halloran, M.C. (2007). Semaphorin3D regulates axon axon interactions by modulating levels of L1 cell adhesion molecule. The Journal of neuroscience : the official journal of the Society for Neuroscience *27*, 9653-9663.

You, Y., Fu, Y., Li, L., Zhang, Z., Jia, S., Lu, S., Ren, W., Liu, Y., Xu, Y., Liu, X.*, et al.* (2024). Systematic comparison of sequencing-based spatial transcriptomic methods. Nature methods *21*, 1743-1754.

Yu, D., Sul, J.H., Tsetsos, F., Nawaz, M.S., Huang, A.Y., Zelaya, I., Illmann, C., Osiecki, L., Darrow, S.M., Hirschtritt, M.E.*, et al.* (2019). Interrogating the Genetic Determinants of Tourette's Syndrome and Other Tic Disorders Through Genome-Wide Association Studies. The American journal of psychiatry *176*, 217-227.

Yuan, W., Ma, S., Brown, J.R., Kim, K., Murek, V., Trastulla, L., Meissner, A., Lodato, S., Shetty, A.S., Levin, J.Z.*, et al.* (2022). Temporally divergent regulatory mechanisms govern neuronal diversification and maturation in the mouse and marmoset neocortex. Nature neuroscience *25*, 1049-1058.

Zhang, M.J., Hou, K., Dey, K.K., Sakaue, S., Jagadeesh, K.A., Weinand, K., Taychameekiatchai, A., Rao, P., Pisco, A.O., Zou, J.*, et al.* (2022). Polygenic enrichment distinguishes disease associations of individual cells in single-cell RNA-seq data. Nature genetics *54*, 1572-1580.
